# Supplementary material for: Are carbon emissions trading and green financial instruments synergistic? -Comprehensive quantitative research based on content analysis
Source: PLoS One. 2024 Mar 7;19(3):e0298601. doi: 10.1371/journal.pone.0298601 (PMC10919601; doi:10.1371/journal.pone.0298601)
Supplement: S1 Data — (ZIP) [file pone.0298601.s001.zip › Data/地级市Z_F工作报告.zip!/║┌┴·╜¡/╞δ╞δ╣■╢√╩╨/└┤╫╘╣1⁄2╓┌║┼í╛┬φ┐╦╩2╛▌═°í┐_2015.docx]

各位代表：

　　现在，我代表市人民政府，向大会作工作报告，请予审议，并请各位政协委员提出意见。

　　一、2014年工作回顾

　　过去的一年，面对国内外经济下行压力较大的严峻形势，在市委的正确领导下，我们坚持深化改革、稳中求进的总基调，坚定信心，冷静应对，创新举措，把握节奏和风险控制，全力以赴上项目、调结构、促改革、保增长、惠民生，实现了经济平稳发展和民生持续改善。全市实现地区生产总值1238.8亿元，同比增长5.2%。公共财政预算收入74.6亿元，增长15.8%。全社会固定资产投资755.1亿元，增长4.8%。社会消费品零售总额618.4亿元，增长13.5%。城镇、农村居民人均可支配收入分别为21352元和11282元，增长12%和13%。居民消费价格上涨1.3%。

　　——产业项目扎实推进。全市共实施千万元以上产业项目567个，完成投资388.1亿元。其中，固定资产投资5000万元以上产业项目294个，新增57个；超亿元项目174个，新增86个；年度开复工项目282个，已投产项目142个；完成投资296.6亿元，为计划的123.9%。继续把引进项目扩增量、生成项目活存量作为重中之重，精心组织参加港粤招商活动周、民企龙江行、央企龙江行、中俄博览会等经贸招商对接活动26次，签订5000万元以上招商项目协议146个，实际到位资金155.9亿元，其中10亿元以上项目13个,台商投资项目4个。奥地利AST、广东恒大粮油、台湾捷邦、香港义华国际等企业的投资项目落户我市。成功举办首届中国（齐齐哈尔）天然苏打水产业发展论坛。齐齐哈尔绿色食品特色产业园开工建设，市高新区被确定为第一批国家低碳工业园区试点。全市各类产业园区发展到20个，新入驻企业92户;基础设施建设完成投资11.6亿元，增长6.4%；入驻企业实现主营业务收入598亿元、工业增加值184.6亿元，分别增长4.2%和5.6%。

　　——现代农业健康发展。农业生产喜获特大丰收，粮食总产实现“七连增”。积极应对国内外农产品市场变化，抓机遇、调结构、促增收，实施水田开发三年攻坚战，水稻、杂粮分别增加71.7万亩和9万亩，玉米减少109.7万亩。以加快生态、绿色（有机）食品基地建设为重点，新增绿色食品基地面积120万亩。“2513”高产创建工程核心区大垄种植面积增加149万亩。新建县乡村三级农业科技园区158个。全市绿色食品加工企业新增14户，农产品加工转化率达42%，提高1.5个百分点。新建存栏奶牛2000头以上现代示范牧场16个，标准化规模养殖高产优质奶牛达到9万头，位列全省首位。以两大平原现代农业综合配套改革试验区建设为契机，积极推进农村四项改革，为国家和省提供了示范和经验。新增省级现代农机合作社规范社23个，占全省新增总数的46%。土地确权登记试点工作全面启动，土地流转服务平台实现全覆盖。建立注册资本金5000万元以上的融资担保平台17个，创新农村金融产品22种，新增受益农户5.4万户；发放“四权”抵押贷款33.6亿元，余额25.1亿元，增加8.7亿元。整合重组国有粮食购销企业10户，引进资金30.4亿元，新增仓储能力357.5万吨。以市场为导向，整合农村职业教育专业34个，培训农民10万余人次，转移农村劳动力117.3万人，实现劳务收入122.5亿元。治理水土流失面积40万亩，修复水毁工程项目80项。争取土地整治资金3亿元，实施旱改水和建设高标准基本农田33.8万亩。投资7.11亿元，新增高效节水灌溉面积70.3万亩。投资1.28亿元，解决农村18.85万人饮水安全问题。扶贫开发整村推进64个，10万人实现脱贫。美丽乡村建设成效明显，52个省级文明达标村通过验收，为计划的1.6倍。

　　——工业经济逆势前行。规模以上工业完成增加值262.3亿元，增长2.6%。其中，装备制造业产值下滑21.8%；食品产业发展势头强劲，实现产值443.2亿元，占工业总产值的45.1%，超过装备制造业19.9个百分点。28个工业项目列入省“双百工程”。县域工业不断壮大，实现总产值428.4亿元，增长24.3%。认真落实国家和省促进工业经济发展的优惠政策，积极搭建央企对接、对外推介和银企对接平台，累计向上争取资金1.1亿元，46户企业获得助保金贷款4.6亿元。继续实施“小巨人扶持培育计划”，发挥中小企业服务中心作用，积极提供融资担保、项目对接、资本引进、信息咨询等方面服务，帮助中小企业解决历史遗留的房产证和用地手续补办等问题，使80户企业增加融资信用额度48.4亿元，新增规上工业企业44户。非公经济实现增加值664.6亿元，增长7%，占GDP的55.5%。新认定国家级高新技术企业5户、省科技型中小企业65户，科技成果转化57项，高新技术产业增加值占GDP的13%。

　　——第三产业活力提升。第三产业实现增加值531.3亿元，增长6%。万力皮革城、国际五金建材城等项目建成运营。全力推动电子商务发展，我市绿色（有机）食品官方旗舰店在全省率先落户京东商城。成功举办绿博会、小交会等40个展会。我市至韩国釜山、清州以及国内大连、济南、深圳等航线成功开通，增加了至北京、上海航班密度，进出港人数增长25.8%，增速居全省各支线机场首位。龙沙动植物园、鹤之汤温泉等项目投入运营，绿色食品展示交易中心、韩国信友集团音河风景区等项目成功签约。新晋4A级景区3个、3A级景区2个。全市金融机构各项存款余额1434.7亿元，贷款余额1164.3亿元，分别比年初增加141.4亿元和256.1亿元，其中工业贷款余额219.6亿元，中长期贷款余额454.2亿元，分别增长28%和21%。兴业银行、哈尔滨银行克山支行完成批筹，浦发银行、润生村镇银行开设分支机构。不断扩大对外开放，加强与日本、韩国、新加坡、美国、俄罗斯及港澳台等国家和地区交流合作，与美国纽卡斯尔市、俄罗斯乌法市、韩国龙仁市签订友好城市协议。外贸进出口总额实现9.1亿美元，增长10%。

　　——城乡环境治理成效显著。制定出台了《齐齐哈尔市城乡规划条例》，“三化”规划逐步完善。中心城区景观亮化、沿江景观带、劳动湖沿岸、工人文化宫区域综合改造等10个专项规划全部完成。全市改造建设城镇道路165公里、285万平方米，覆盖裸露地面24万平方米，维修巷道82公里，改造标准化公交站点425个。昂齐公路、绥满高速卜奎互通工程和滨洲铁路、讷河市区2座公铁立交桥交工通车。10个省级百强试点镇建设项目70个，完成投资12.9亿元。中心城区改造新建道路72条，亮化升级2座跨江大桥、7个出城口、19条主次干道和85条无灯路，安装路灯6032基；新城主路网及基础设施配套基本建成，高铁站及配套附属建设按期完成；劳动湖沿线桥梁全部实现通行，南扩工程全线贯通并开闸通水。全市“三供三治”项目新开工40项，完成投资13.5亿元，铺设各类管网340公里。中心城区供热改革启动实施，由大型企业带动的区域供热格局初步形成。全市城市管理“四级网络”全覆盖，拆除违章建筑7071处、围栏2.8万延长米、棚亭751处，清理绿化带超高土16.2万立方米。全市淘汰黄标车及老旧车辆22678台，新增清洁能源公交车辆116台，城区撤并整合燃煤小锅炉130台。城区栽植树木130万株，新增绿地24万平方米；乡村植树造林25万亩。创建省级生态县4个、国家和省级生态乡镇10个、省级生态村162个。全面完成节能减排示范市建设和主要污染物减排年度任务，万元GDP综合能耗下降5.9%，空气质量国家二级以上标准天数达315天。

　　——人民生活明显改善。年度33件民生实事完成29件，其余4件由于规划设计、缓解交通、改造道路和市场化改革等原因调整了时间进度，今年继续推进落实。城乡就业一体化改革取得重大突破，城镇新增就业11.8万人，城镇登记失业率控制在4.1%。医疗保险管理和待遇水平有新提高，居民养老保险覆盖面进一步扩大。新农合参合率达到99.68%，人均筹资额达到390元。按政策提高了企业离退休职工基本养老金标准。环卫工人月工资增加200元。拖欠多年的“四类”企业省级以上劳模待遇全部兑现。提高了社区工作者补贴标准。城乡低保应保尽保，保障标准高于省定标准且地方财政补助水平全省最高。低保和低收入失能半失能老人护理补贴标准超过省标一倍，处于全省领先水平。五保供养达到省级标准。全市开发各类商品房507万平方米；开工建设棚改回迁房1.87万套，基本建成1.44万套；建设公租房、廉租房2397套，14万平方米；改造农村泥草（危）房5.2万户，新建、续建灾后房屋2771户。全市既有建筑节能改造357万平方米，中心城区改造老旧小区楼房343栋。全市创建省级以上物业示范项目5个，中心城区318栋无物业管理楼房的物业服务问题基本解决。全市新改扩建公办幼儿园17所，建设义务标准化学校31所，农村中小学校“小火炉”改造全部完成。投入1240万元，为全市429所学校安装护眼灯49982盏。在全省率先开展职业教育改革试点市创建工作。市图书馆新馆续建完成并正式开馆。扶持市马戏团、话剧团等文艺院团创作精品剧目和常态化演出，动画片《神鹤丹丹》登陆央视。举办了第二届国际鹤文化艺术节、扎龙国际婚礼文化节和东北三省第三届合唱节，我市成为东北三省首家“中国合唱基地”。成功晋升为国家历史文化名城。市文广新局荣获我省唯一全国文化系统先进集体。创编并推广“鹤城鹤舞”有氧健身操，新增全民健身场地350处，建成中心村文化设施166个。作为国内唯一参赛城市，成功承办亚洲冰球联赛。推进医疗一体化改革，市带县医联体达到100%，县乡一体化率达到99%。出台鼓励社会力量兴办养老服务机构的扶持政策，签约养老服务业项目8个，市养老护理综合实训基地开工建设，新建100张床位以上民营养老机构5家，被确定为全国养老服务业综合改革试点城市。“双拥”工作扎实开展，重点优抚对象“三难”和退役士兵安置问题得到有效解决。新创建城市示范社区8个、农村示范社区16个，城市社区公益用房面积平均达到790平方米。改革完善食品药品监管体系，蝉联中国十佳食品安全城市。加强社会治安综合治理，不断加大案件侦办力度，城区重点部位监控全覆盖，平安城市建设成效显著。加大安全生产联合执法力度，消除了一批重大和较难整改隐患，安全生产形势总体向好。畅通信访渠道，省市县三级视频接访实现互联互通，一批信访疑难案件得到化解。

　　——政府自身建设不断加强。扎实开展党的群众路线教育实践活动，认真贯彻落实中央八项规定和省九项规定，坚决反对“四风”，全市清理腾退办公用房6.3万平方米，清理各类“吃空饷”人员768人，新建楼堂馆所零审批，“三公经费”下降30%。启动实施新一轮政府职能转变和机构改革，事业单位分类改革扎实推进。加大简政放权力度，市级审批事项精简67.5%，县（市）区精简30%。优化审批流程，组建“中介超市”，全面实施建设工程项目并联审批，审批服务效率大幅提高。市县乡村四级政务服务体系全覆盖，电子监察系统实现市县乡三级联网，市公共资源交易中心功能进一步扩充，县（市）交易中心实现试运行。成立市级企业投诉中心，畅通了保护企业和投资者合法权益渠道。在全省率先放宽注册资本登记条件。自觉接受人大和政协监督，人大代表议案和政协委员提案办结率均达100%。起草审修地方性法规草案3部，制定政府规章8部。深入开展普法教育，荣获全国“六五”普法中期先进城市。强化行政监察和审计监督，曝光了一批典型案件，监督审查政府投资项目209个、节约资金3.1亿元。

　　机关事务、外事侨务、民族宗教、新闻出版、社会科学、妇女儿童、统计监测、防灾减灾、残联、地方志、人防、档案、气象、地震等领域也都取得了新成绩。

　　各位代表！过去一年的成绩来之不易。这是省委、省政府和市委正确领导的结果，是市人大、市政协及社会各界有力监督、鼎力支持的结果，是全市人民团结一致、努力拼搏的结果。在此，我代表市人民政府，向全市人民，向驻齐解放军、武警官兵，向所有为我市经济社会发展作出贡献的各界人士，致以崇高的敬意和衷心的感谢！

　　同时，我们也清醒地看到，全市经济社会发展和政府工作中还存在一些问题。主要矛盾仍然是发展不足，调结构、 促改革、保发展、惠民生的任务艰巨，突出表现为：一产基础不牢，产业化程度低；二产结构不优，市场竞争力弱；三产活力不强，拉动作用小。牵动发展的大项目、好项目不多，对财政贡献率较低；城乡居民收入水平不高，城市基础设施欠账较多；财政收入与民生支出的矛盾突出，惠民生能力与群众期望差距大；县域经济总量偏小，工业化、城镇化进程缓慢；政府职能转变与经济发展需求差距较大，政府工作的专业化、法治化水平还需提高；庸、懒、散、慢等现象还不同程度地存在，改作风、反“四风”任重道远。对此，我们一定高度重视，认真加以解决。

　　二、2015年工作任务

　　2015年，是深入落实党的十八届三中、四中全会，省委十一届五次全会和市委十二届六次全会精神的重要一年，也是积极适应经济发展新常态、全面推动改革发展的关键之年，更是全面完成“十二五”规划的收官之年。全市经济社会发展既面临诸多困难和挑战，也具备很多有利条件和积极因素。一方面，虽然世界经济增速可能略有回升，但总体复苏疲弱态势难有明显改观，仍处于深度调整中。国内经济发展进入新常态，结构调整阵痛显现，下行压力较大。我市装备制造、冶金、化工等传统行业生产经营异常艰难的状况短期内还很难改变。另一方面，国内经济发展长期向好的基本面没有变，改革创新动力不断激发，内需潜力巨大并逐渐释放。基础设施互联互通和一些新技术、新产品、新业态、新商业模式的投资机会大量涌现。随着国务院稳增长19条、支持东北振兴35条及我省稳增长65条等一系列宏观调控政策的具体实施，经济社会发展必将呈现稳中有进、稳中向好的新格局。珠三角、长三角的IT产业正在向内地和区位更加优越的地区转移，特别是我省“五大规划”和“龙江陆海丝绸之路经济带”发展战略的实施，我市入选国家新型城镇化试点城市，绿色（有机）食品资源优势和潜能正在逐步释放，以及俄罗斯经济发展对高端机电产品和绿色（有机）食品需求的快速增长，都将为我市带来新的发展机遇，也必将有力推动全市“三化”建设。只要我们牢牢把握各种有利机遇，解放思想，坚定信心，趋利避害，凝心聚力，创新求实，开拓奋进，就一定能够实现全市经济社会平稳较快发展。

　　今年政府工作的总体思路是：深入贯彻党的十八届三中、四中全会和中央、省委经济工作会议精神，主动适应经济发展新常态，抢抓国家实施“一带一路”和我省实施“五大规划”、“龙江陆海丝绸之路经济带”等战略机遇，按照市委十二届六次全会总体部署，以“三化”建设为统领，以项目建设为抓手，统筹推进结构调整、产业提升、深化改革、扩大开放、城乡治理、改善民生等重点任务，增强政府推动经济发展和依法治理社会能力，奋力开创全市经济社会发展新局面。

　　主要预期目标是：全市地区生产总值增长6.5%左右，公共财政预算收入增长7.4%左右，规模以上工业增加值增长3%以上，全社会固定资产投资增长10%以上，社会消费品零售总额增长13%以上，城镇、农村居民人均可支配收入分别增长9%和10%以上，城镇登记失业率控制在4.5%以内，城镇化率提高1.5%，万元GDP综合能耗下降5.9%。

　　为实现上述目标，重点做好以下工作：

　　（一）全力以赴上项目，加快夯实区域经济实力。深入实施项目建设牵动战略，重点谋划建设固定资产投资超5000万元、年度投资超1000万元的产业项目260个以上。立足资源优势、区位优势和历史文化禀赋，围绕做大增量、盘活存量、提高质量上项目。既注重谋划建设一批支撑经济发展、促进产业结构调整的重大项目，也注重加快建设一批促进创业就业的中小项目，并在现有项目裂变生成上下功夫。以市场为导向，引导组织各县（市）区开展区域合作，整合资源，形成合力，走产业化、专业化发展道路，培育主导产业和产品，形成“一县多品”、“一品多县”的发展态势。坚持从我市拥有、国家支持的特色优势产业项目做起，切实抓好主导产业全链条的延伸，形成各县（市）区分工协作、产业聚集的发展格局。在园区建设上，改变过去独立、分散的厂区布局，把基础设施建设、产业项目引进、工业物业管理和发展生产服务型企业统筹规划和运作，集约利用资源，增强园区对项目的孵化力、承载力和吸引力，促进相关联的产业项目集聚集群发展。充分利用国际国内两个市场、两种资源和两类规则，把握市场规律，紧跟市场导向，适应市场需求，抓好项目谋划和引进，推动绿色食品、IT等项目基地建设。紧盯已有经贸招商活动的信息和线索，深入开展洽谈合作，促进有意向的项目尽快签约落地。发挥各级干部和专业团队能动作用，建立调度保障、推进服务、承诺问责等制度，对项目报批、征地、开工、建设、投产、达效等实行全程跟踪服务，力争固定资产投资5000万元以上项目建成投产80个，达产达效110个。

　　（二）转变方式促增收，全面发展现代化大农业。紧紧围绕两大平原现代农业综合配套改革试验任务，以农业稳产、农民增收、农村发展为目标，全面推进现代农业发展。以国内外市场需求及国家政策调整为导向，进一步调整优化种植结构，新增水田面积100万亩。发挥农业科技园区示范带动作用，扩大水稻、马铃薯等优良品种高产栽培模式推广范围。搞好绿色（有机）食品基地建设，加快农产品及加工食品与国际检验检测标准接轨，推动我市大宗农产品、特色加工食品及果蔬类农产品进入国际市场。办好苏打水、马铃薯等产业发展论坛，强化品牌整合，提高市场整体竞争力。以物联网为载体，搞好绿色（有机）食品营销，并与各家电子商务平台对接，全力打造以我市为中心的绿色（有机）食品商务平台，加快建成全国的优质农产品、有机绿色食品的生产中心和集散中心，促进绿色食品生态价值与产地品牌的双增值。以“两牛一猪”为重点，实施畜牧业全产业链发展模式，新增标准化规模饲养奶牛3.6万头、高档优质肉牛1万头、普通肉牛10万头，鼓励推广“龙江雪牛”繁育；以金锣生鲜肉出口俄罗斯市场为突破口，提高生猪规范养殖标准和技术，扩大养殖规模，增加出口份额；加快肉类加工企业发展，推动台湾嘉一香生猪养殖屠宰加工等项目早日达产。以克山仁发、昆丰，龙江超越，依安象屿等合作社为示范，以效益为根本，以增收为目标，务实有效地鼓励支持发展各种形式合作社。扎实推进土地确权登记试点工作，完善农村土地流转平台建设，适应农业农村发展新模式。继续深化农村金融改革，加强农村信用体系建设，加快提高农业农村信贷融资能力。积极引进战略投资主体和社会资本，着力推进粮食购销企业改革，开展农民合作社政策性粮食收购试点。以劳动力技能提高和适应市场需求为根本，推进省部共建现代农村职业教育改革试验区建设，转移农村劳动力120万人，实现劳务收入130亿元。加大农资、农产品等市场监管，确保农副产品质量安全。加强农业生态环境保护和修复，增施生物有机肥料，减少农药化肥使用量，探索秸秆综合利用新途径。抓住国家加大对我省三江治理投资的机遇，积极推进水利建设与管理体制改革，加快完善农田水利基础设施；通过土地整治，建设高标准基本农田25万亩，新增高效节水灌溉面积60万亩；实施中小河流治理项目3个，消险加固小型水库3座。严格水资源管理，解决农村20.1万人饮水安全问题。实施精准扶贫，加大革命老区和少数民族区域扶贫力度，农村贫困人口减少10万人。

　　（三）坚持不懈调结构，促进工业经济提质增效。充分利用国内外经济深度调整的机遇，优化工业结构，加快转型升级，着力促进我市大型龙头企业稳定发展、中小企业做强做大形成规模、小微企业发展活力竞相迸发。加大对传统装备制造、石化、冶金等龙头企业在开拓市场、就地配套、产业集聚、分离办社会等方面的服务支持力度，扎实推进国有企业和厂办大集体改革，鼓励支持具备条件的国有企业实现产权多元化和混合所有制，完成中直企业“三供一业”分离移交后续工作，增强企业内生动力。装备制造业主营业务收入增长5%以上。围绕水、乳、豆、薯、酒等九大产业，在产、加、销等环节实行全链条扶持，加快绿色食品产业发展。食品加工业主营业务收入增长10%以上。大力扶持新能源、新材料、新生物等新兴产业发展，加快培育工业经济新的增长点。以科技转化为支撑，加快发展科技服务业，切实加强与大专院校、科研院所的合作，推动齐大、江南大学、东北农大、中国农大等高校在我市建立产业技术研究院，促进中小企业技术、管理和销售创新驱动发展。高新技术产业增加值占GDP的11%以上。完善《关于加快中小企业发展的意见》，建立企业发展会商制度，重点解决历史遗留、现实存在和发展预判等方面的问题，营造优良发展环境。依托中小企业服务平台，积极鼓励引导中小企业通过各类资本市场提高发展能力，加快推进企业股权交易、新三板挂牌上市和企业集合票据发行等工作，着力解决企业融资难题。以促进全民创业就业为目标，充分发挥融资担保平台、助保金贷款的作用，支持小微企业发展，形成全民创业氛围。采取走出去、请进来等方式，加强对民营企业创业者、经营者培训，提高企业家素质和创业能力。

　　（四）提升功能强辐射，推动第三产业蓬勃发展。紧紧围绕增强区域中心城市辐射和带动功能，在巩固传统服务业的基础上，全力推进现代服务业发展。完成万达广场、百花小商品交易中心等项目建设，全面加快铁锋综合物流园、象屿粮食仓储等项目进度，着力打造区域商品物流集散交易中心和基地。加快发展电子商务，整合培育一批具有本地特色的品牌电商和领军企业。加快推进东北亚北方商品交易中心建设，提高绿博会、小交会等品牌展会的层次和水平。搞好旅游发展战略规划，推进温泉小镇、扎龙湿地、奥悦室内滑雪及嬉水乐园、碾子山滑雪风情小镇、扎龙房车营地等重点旅游项目建设。深化“4+1”城际旅游联盟合作，旅游收入增长12%以上。积极引进各种金融机构，鼓励金融机构优化信贷结构，支持实体经济发展。加大改革力度，促进教育、卫生、文化、体育、养老等领域资源共享、人才共用、融合发展，激发城市发展内生动力和第三产业发展活力。认真贯彻中央文艺座谈会精神，扶持各类文化组织蓬勃发展，重点支持马戏团、话剧团等院团创作文艺精品，鼓励文艺工作者深入基层、服务群众，推动公民文明素质和城乡文明程度“双提高”，逐步让文明成为一种习惯，不断提升城市文化软实力。继续办好国际鹤文化艺术节和扎龙国际婚礼文化节，举办金长城遗址历史文化高端论坛。广泛开展与俄罗斯、韩国、新加坡、澳大利亚等国家和地区的文化交流合作。坚持教育优先发展，着力办好人民满意教育。建立人才共享机制，让更多优秀的艺体工作者进入校园发挥特长，提升中小学生综合素质。积极承办国内外重大体育赛事，打造冰雪重大赛事活动基地，引领和带动体育产业发展。推进黑龙江省西部区域医疗中心建设，支持市第一医院等医疗机构引入战略投资者，实施多元化办医改革发展，打造医疗服务综合体和专业特色医疗基地。深入开展爱国卫生运动，提高群众生活质量。大力发展养老产业，鼓励扶持机构养老发展，探索居家养老服务新途径，打造全国候鸟式养老基地，带动就业创业及相关产业发展。完善城乡社区建设管理服务体制机制，统筹用好各类人才、管理、服务等资源，推动政府公共服务及管理职能下沉。积极培育发展社会公益组织，大力培养社会工作人才，提高社会化服务水平。推动军民军地融合发展，争创全国双拥模范城“七连冠”。

　　（五）内引外联促开放，不断拓展发展空间。发挥我市作为“龙江陆海丝绸之路经济带”重要节点城市的优势，结合俄罗斯相关产品进口需求扩大的实际，大力引进外向型贸易服务及加工企业，加快启动综合保税区申建工作，积极搭建对俄、对欧经贸合作平台，提升面向俄罗斯、联系欧亚的桥头堡承载能力，逐步扩大进出口贸易份额。充分利用本地劳动力等生产要素成本低，特别是中东铁路对俄欧产品输出运期短、运费低等优势，积极承接发达地区产业转移，建设国家级产业转移承接示范区、黑龙江省西部对俄欧进出口加工基地和国际货物仓储集散中心。加快畜牧养殖和果蔬出口基地建设，借助黑河、满洲里等口岸扩大对俄出口。在注重招商的全局性、整体性和信息资源共享、项目落地统筹的基础上，瞄准珠三角、长三角、台湾等重点区域和国内外大企业及各类商会协会，开展定向式、定点式、链条式、集群式招商，力争新引进签约5000万元以上项目150个，到位资金165亿元。全面启动和深入推进IT、机电装备、中小企业配套、绿色食品、农机、畜牧、果蔬和商品集散等产业园区建设，积极招引相关领域的领军企业入驻园区。启动机场扩建工程，完善航线网络，加密至广东航班，力争开通至福建、浙江等发达省份和俄罗斯、日本等国家的航线。积极推动与德国、澳大利亚、新西兰、日本、冰岛、奥地利等国家的城市建立合作关系，在职业教育、文化体育交流、畜牧业发展、温泉养生、旅游资源开发等方面加强合作，提升对外开放度。

　　（六）统筹兼顾抓治理，大力改善人居和生态环境。启动新型城镇化试点城市建设，推动城市、城镇和新型农村社区协调发展。中心城区在抓好老城区改造建设的同时，推动IT产业和高新技术产业向新城集聚，推进休闲、娱乐、教育、医疗、交通等公共服务向新城延伸，汇聚更多的人流、物流、资金流和信息流，加快产城一体化发展和城市化进程。着力保障哈齐客运专线调试运行和新城公路客运枢纽投入运营，推进南互通、南外环、江桥黑蒙公路、北安至富裕高速公路、滨洲铁路电气化改造、齐北铁路复线等项目开工建设，构建辐射东北地区西北部的现代化交通体系。以满足群众需求和城市发展运行需要为重点，进一步完善城镇路网并加大维护力度，加快全市“三供三治”、电力通讯等基础设施建设，推进供水、供热、供气、污水处理、环卫保洁和垃圾处理市场化运营，提升监管和服务水平。统筹搞好商品房开发和保障房建设，促进房地产业稳定健康发展。建立科学规范的城市管理体系，加强城乡环境综合治理，提高环卫机械化作业率，中心城区机械化清扫率达到70%以上。以创建国家级园林城市为目标，提升城区绿化水平，新植树木140万株，新增绿地26万平方米。因地制宜、灵活多样地推进美丽乡村建设，着力体现地方特色和文化内涵；创新乡村管理方式，实行村容村貌管理常态化；抓好县乡主要道路两侧、农村水渠周围绿化，植树造林10万亩。加快10个省级百强试点镇建设，建成一批全省新型达标（示范）村。加大水土流失治理力度，新增治理面积40万亩。加强湿地保护管理。完成富裕县国家级生态县创建工作，加大国家、省级生态乡镇和生态村创建力度。以加快节能减排示范市建设为契机，探索生态城市建设新途径，大力实施大气污染防治三年行动，突出抓好嫩江流域水污染防治，严格控制主要污染物排放。二氧化硫、氮氧化物分别削减2.81%和3.43%，可吸入颗粒物(PM10)浓度比2012年降低4%，空气质量国家二级以上标准天数超过275天。

　　（七）全心全意惠民生，努力增进全市人民福祉。坚持心怀群众、情系人民，集中力量办好30件民生实事。（1）实施全民就业技能培训工程和高技能人才培养计划，就业技能培训8万人，岗位技能提升培训3万人，高技能人才培养8000人，城镇新增就业9万人。（2）开展城乡居民大病医疗保险，减轻群众就医负担。（3）继续提高社区工作者生活补贴标准，并落实“三险一金”待遇。（4）为全市环卫工人增加人身意外伤害保险，建立全市养老机构入住老年人意外伤害保险制度。（5）提高全市福利机构“三无”人员集中供养待遇标准。（6）扩大法律援助范围，降低准入门槛，办理法律援助案件1400件以上。（7）开展精神病救助，为贫困精神患者提供基本治疗及药品补贴救助，为贫困重度精神病患者提供3个月的免费住院医疗。（8）实施残疾人关爱工程，对中心城区10处公共服务场所和300户贫困残疾人家庭进行无障碍改造；为全市升入高等院校的特困残疾考生及贫困残疾人家庭子女提供一次性助学补助，为新建社区（村）残疾人康复站发放康复训练器材28套，为1000名贫困残疾人免费配发助视器、助行器和助听器。（9）解决全市中小学生在校饮水难问题。（10）扶持发展全市民办幼儿园，着力提高师资水平，全市培训民办幼儿教师600名。（11）整合培训资源，丰富内容，集中在市新图书馆开办“鹤城讲坛”，面向市民举办各类讲座，打造公益性学习交流平台。（12）发挥“中国合唱基地”作用，办好第十七届中国老年合唱节。（13）深入实施文化惠民工程，打造“鹤城百姓文化季”等群众文化活动品牌，以购买公共服务的方式，实施精品剧目常态化演出200场次以上。（14）启动实施工人文化宫区域综合改造工程。（15）举办齐齐哈尔市首届“动物冰雪大世界”。（16）筹办冬季国际冰雪马拉松，增强我市体育赛事影响力。（17）推广“鹤城鹤舞”有氧健身操，打造全民健身活动新名片。（18）改善城乡居民出行环境，全市维修改造巷道60公里；中心城区实施南浦、永安立交桥加固维修和嫩江北部公路大桥维修改造工程；妥善解决两座嫩江公路大桥收费问题，使嫩江两岸、中心城区与西部县区交通更加便捷顺畅。（19）全市改造低温楼200万平方米；利用三年时间，对全市现有老旧楼房外墙和楼梯间进行粉刷，今年分别粉刷220万平方米和95万平方米；利用两年时间，对全市需改造的漏雨屋面、供水和排水管线进行改造，今年分别改造62万平方米、31万延长米和20万延长米。（20）推进供热设施升级改造，全市撤并整合燃煤小锅炉179台，改造老旧供热管网192公里。（21）全市清理绿化带超高土18万平方米左右,绿化、硬化裸露地面30万平方米左右，拆除违章建筑4500处左右。（22）对龙沙、东湖、鹤城、金盾4个公园进行维修、改造和升级建设。（23）在中心城区主干道两侧新建公厕20座，鼓励和引导沿街单位开放室内厕所。（24）完成黎明、南山和红星生活垃圾填埋场搬迁。（25）实施废品收购经营、废塑料再生加工、废机油收集处置等影响居民生活环境企业清理整顿行动，建设专业化园区，加快推动“三废”企业和噪声污染的小加工厂迁出城市居民区。（26）打造“公交都市”，制定公交发展专项规划，全市新增40辆新能源公交车，对全部公交站亭进行亮化，中心城区建成5处公交首末站。（27）中心城区新增临街停车场22处。（28）实施食品安全示范工程，在中心城区创建省级餐饮食品安全示范店，确保“舌尖上的安全”。（29）成立“12345”便民服务热线受理中心，将29个市直部门和“非应急类”便民服务热线全部整合其中，方便群众投诉咨询。（30）深化平安鹤城建设，加快构建应急管理平台，提升城市管理信息化水平，提高突发事件预防预警和应急处置能力；完善立体化社会治安防控体系，全面落实安全生产“四级五个全覆盖”，着力保障人民群众生命财产安全。

　　（八）改革创新提效能，着力建设人民满意政府。面对复杂的宏观经济环境和繁重的工作任务，我们必须加强政府自身建设，切实做到诚信守诺、履职尽责，改革创新、高效服务，依法行政、廉洁自律。巩固和发展好党的群众路线教育实践活动成果，坚决把各项整改措施落实到位，着力健全作风建设长效机制。推行政府法律顾问制度，完善科学决策程序和参与机制，建立行政机关内部重大决策合法性审查、终身责任追究和责任倒查制度，实现科学民主决策。从政府组成人员和工作部门做起，带头学习、模范遵守宪法和法律，不断提高依法行政水平。坚持问题导向，继续清理行政审批事项和具有审批性质的管理事项，与全省同步建成网上审批系统，探索建立审批服务代办制，完善市县两级公共资源交易中心建设，发挥好企业投诉中心作用，坚决查处损害经济发展的人和事，进一步优化经济发展环境。深化市县（区）两级政府行政执法管理体制改革，规范行政自由裁量权标准，推进行政执法和收费平台建设，探索建立执法全过程记录制度，提高执法效能和规范化水平。抓好事业单位分类改革的相关配套工作。积极适应经济发展新常态，科学编制好“十三五”规划。坚决执行人大及其常委会决议决定，自觉接受人大法律和工作监督、政协民主监督以及社会各界监督。充分发挥预算刚性约束作用，将所有政府收入和支出全部纳入预算管理，改进购买公共服务的方式和机制，强化财政资金使用绩效评价考核，把有限的资金用在群众最期盼、发展最需要的地方。严格落实党风廉政建设责任制，保持正风肃纪高压态势，加强对经济责任、重要事项及民生项目的监督审计，强化工作督办落实和效能问责，全面推动政府工作高效廉洁运转。

　　各位代表！齐齐哈尔正处于加快发展的关键期和全面深化改革的攻坚期，任务艰巨，责任重大，使命光荣。全市各级政府工作人员都要忠诚于党和人民，为全市经济社会发展和民生持续改善尽职尽责。让我们在市委的正确领导下，解放思想，抢抓机遇，万众一心，奋力谱写全市改革发展的新篇章，齐齐哈尔的明天一定会更加美好！
